# Bromo-Chloralum in Scirrhous Affections of the Stomach

**Published:** 1873-07

**Authors:** X. T. Bates

**Affiliations:** New Lebanon, N. Y.


ARTICLE IV.—*Bromo-Chloralum in Scirrhous Affections of the Stomach.* By X. T. BATES, M.D., New Lebanon, N. Y.

I desire to call the attention of the profession to the use of bromo-chloralum manufactured by Tilden & Co., simply as a palliative measure in cancer of the stomach, in which affection it has very recently proven, in my hands, of signal service in the case of M. B.,—male—Irish—farmer.

At the period of my first visit, in January, of the present year, I learned that he had been on the decline for a year or more, and at times a great sufferer, with progressive loss of strength and weight, until he was finally obliged to take to his bed altogether. The history and condition of the case were such as to leave no doubt as to the cause of his sufferings and of the ultimate result. I found him anæmic and emaciated, appetite impaired, with marked feebleness and lack of vital force, lancinating pains in the vicinity of the stomach, cancerous cachexia and occasional vomiting of purulent matter, with eructations so offensive as to demand imperatively some combative agent.

Having on several previous occasions satisfactorily tested the disinfecting virtues of bromo-chloralum in the sick room, and also demonstrated its unquestionably remedial properties in foul breath, it occurred to me to make trial of it in this case—prescribing as follows:

R. Bromo-Chloralum, - - - - - - - - dr. i.
Water, - - - - - - - - - - - oz. i.
Ess. Wintergreen, - - - - - - - - q. s.
Sig. One teaspoonful every four hours.

The effect was magical; the offensive factor at once disappeared, the nausea was controlled, the countenance became brighter, and for a short time the hopes of the patient were revived that ultimately he might be cured. The bromo was the only medicine that appeared to afford him any relief, and its use was continued up to the time of his death, happily subserving the purpose for which it was given.

